# Fertility of frozen-thawed stallion semen cannot be predicted by the currently used laboratory methods

**DOI:** 10.1186/1751-0147-48-14

**Published:** 2006-08-17

**Authors:** P Kuisma, M Andersson, E Koskinen, T Katila

**Affiliations:** 1 Department of Clinical Veterinary Sciences, University of Helsinki, 04920 Saarentaus, Finland; 2 Department of Animal Sciences, University of Helsinki, PL 28, 00014 Helsingin yliopisto, Finland

## Abstract

The aim of the project was to use current simple and practical laboratory tests and compare results with the foaling rates of mares inseminated with commercially produced frozen semen. In Exp. 1, semen was tested from 27 and in Exp. 2 from 23 stallions; 19 stallions participated in both experiments. The mean number of mares per stallion in both experiments was 37 (min. 7, max. 121). Sperm morphology was assessed and bacterial culture performed once per stallion. In Exp. 1, progressive motility after 0, 1, 2, 3, and 4 h of incubation using light microscopy, motility characteristics measured with an automatic sperm analyzer, plasma membrane integrity using carboxyfluorescein diacetate/propidium iodide (CFDA/PI) staining and light microscopy, plasma membrane integrity using PI staining and a fluorometer, plasma membrane integrity using a resazurin reduction test, and sperm concentration were evaluated. In Exp. 2, the same tests as in Exp. 1 and a hypo-osmotic swelling test (HOST) using both light microscopy and a fluorometer were performed immediately after thawing and after a 3-h incubation. Statistical analysis was done separately to all stallions and to those having ≥ 20 mares; in addition, stallions with foaling rates < 60 or ≥ 60% were compared. In Exp. 1, progressive motility for all stallions after a 2 – 4-h incubation correlated with the foaling rate (correlation coefficients 0.39 – 0.51), (p < 0.05). In stallions with > 20 mares, the artificial insemination dose showed a correlation coefficient of -0.58 (p < 0.05). In Exp. 2, the HOST immediately after thawing showed a negative correlation with foaling rate (p < 0.05). No single test was consistently reliable for predicting the fertilizing capacity of semen, since the 2 experiments yielded conflicting results, although the same stallions sometimes participated in both. This shows the difficulty of frozen semen quality control in commercially produced stallion semen, and on the other hand, the difficulty of conducting fertility trials in horses.

## Background

In many countries, artificial insemination (AI) has superseded natural mating as a breeding method for mares. Use of frozen semen, however, has not gained widespread use in horses, due to low pregnancy rates. In addition to semen quality, many other factors affect the outcome of AI, including the handling and freezing methods of semen, AI dose, timing of AI and management and fertility of the mares [1]. There is considerable variation between individual stallions in how their semen survives freezing and thawing. Otherwise fertile stallions can produce semen that results in very poor post-thaw pregnancy rates [2]. *Tischne*r [3] estimated that approx. 20% of stallions are "good freezers", another 20% are "bad freezers", and the majority of stallions, 60%, produce semen that is affected adversely, but may be freezable using certain techniques. Unlike bulls, stallions are not selected for breeding on the basis of fertility or semen freezability [1]. Therefore, not much progress is to be expected in the use of frozen stallion semen. For prediction of fertility and for improving freezing methods, it is important to develop reliable techniques to assess the quality of semen *in vitro*. Many methods exist and are used, but not many studies have examined or showed the connection between laboratory test results and fertility of frozen-thawed stallion semen [4], [5]. The relationship between motility, the most frequently used test in horses, and fertility is far from clear [6], [7] and particularly for frozen semen it is not an exact measure of fertilizing potential [8]. In *Malmgren*'s review [9], conflicting correlations were reported between morphology – another commonly used test – and fertility of fresh stallion semen. It is often assumed that the condition of spermatozoa surviving after cryopreservation would be similar to the pre-freeze state. There is evidence that also the survivors have been affected [10]. Therefore, assessment and methods of examination applied for fresh semen may not be as useful for frozen semen.

*Amann *[11] stated that establishing a correlation between different attributes of semen and fertility is not sufficient. The goal is to develop laboratory tests that are predictive of fertility, which is not an easy task to achieve, particularly in horses. To determine if a laboratory test is correlated with fertility, one must have specific, precise, and accurate laboratory tests and precise and accurate fertility data from an adequate number of females. Tests of several independent parameters should be made [11-13,7]. *Graham *[14] listed several attributes that a sperm must possess to fertilize an oocyte, including motility, normal morphology, sufficient metabolism for energy production, and membrane integrity. Measurement of only a single attribute will fail to detect sperm defective in a different attribute and will overestimate the number of fertile sperm in the sample.

Obtaining good fertility data is difficult in horses. The number of mares and stallions used is too small, too few mares are inseminated at the appropriate time using adequate AI doses, too few semen samples are evaluated in an appropriate manner from each male, and the fertility data of mares is inaccurate [11].

Sperm membranes are particularly vulnerable during freezing [10]. This suggests that tests evaluating sperm membrane integrity should be used in the evaluation of frozen semen. On the other hand, spermatozoa that have survived freezing and thawing may be a selected subpopulation, which has unusually stable membranes. These membranes may also be unresponsive to physiological stimuli. If this is the case, then cryopreservation process may select viable, but relatively infertile sperm [10]. Membranes of cryopreserved spermatozoa are less able to withstand osmotic stress than fresh spermatozoa [15]. Velocity (curvilinear and mean path velocities) and linearity of cryopreserved spermatozoa are generally reduced [10]. A commonly used selection criterion in commercial stallion semen production is post-thaw progressive motility of ≥ 30–35%.

The aim of the present study was to use economically feasible and simple laboratory tests and correlate them with the foaling rates of mares. The pregnancy rates per cycle would have better reflected fertility [16], but they were not available from all mares. The aim of the study was also to analyze the overall quality of commercially produced semen doses.

## Materials and methods

Results of frozen semen evaluation tests and foaling rates of mares were compared in 2 experiments. In the first experiment, semen of 27 stallions was tested and in the second experiment semen of 23 stallions; 19 stallions participated in both experiments. Only stallions having foaling data from at least 7 mares were included; the data were also analyzed separately for stallions having ≥ 20 mares.

### Frozen semen

#### First experiment

Semen straws, frozen between 1988 and 1997, were available from 27 commercial stallions from Sweden (22), Finland (2), Italy (2), and the USA (1). Twelve of the stallions were American Standardbreds and 15 others represented various breeds of riding horses. Semen from one stallion was frozen in 5-mL straws, from 22 in 2.5-mL straws and from 4 stallions in 0.5-mL straws. The foaling data originated from Finland and Sweden from 1989 to 1998. The mean number of mares per stallion was 37 (min. 7, max. 121). The average foaling rate was 56% (min. 0, max. 86%). Twelve stallions had foaling rates > 60% and 15 had foaling rates < 60%.

#### Second experiment

Semen straws, frozen between 1988 and 1998, were available from 23 commercial stallions from Sweden (18), Finland (3), Italy (1) and Germany (1). Semen from 18 stallions was frozen in 2.5-mL straws and 5 in 0.5-mL straws. Seven stallions were American Standardbreds and 16 were various breeds of riding horses. The foaling data originated from Finland and Sweden from 1989 to 1999. The average number of mares per stallion was 37 (min. 7, max. 121). The mean foaling rate was 60% (min. 11%, max. 86%). Fourteen stallions had foaling rates of > 60% and nine < 60%.

### Experiments

In the first experiment, the semen evaluations were performed once immediately after thawing, but motility assessment using light microscopy was continued for 4 h. Since the incubation appeared to differentiate sperm more readily than examination immediately after thawing, all tests were carried out 0 and 3 h after thawing in the second experiment. Bacterial culture was performed only in the first experiment and the hypo-osmotic swelling test (HOST) only in the second experiment. The HOST was included in the evaluation tools due to promising results in stallions [17,18]. The morphology was assessed from frozen-thawed spermatozoa once per stallion.

### Thawing and incubation

The 0.5-mL straws were thawed at 37°C for 30 sec, the 2.5-mL straws at 50°C for 40 and the 5-mL straws for 45 sec. The semen concentration was measured in a Bürker counting chamber, and the total number of spermatozoa per straw calculated. An insemination dose was one straw when the 2.5- or 5-mL straws were used and from 1 to 10 straws for the 0.5-mL straws. The semen was extended with a warm (+30°C) skim milk extender [19] to a concentration of 20–30 × 10^6 ^spermatozoa/mL.

The sample for the longevity test was prepared by placing 0.5 mL of extended semen into a 3-mL vial enclosed with a cap. The sample was kept in a water bath at 37°C for 4 h (Exp. 1) or 3 h (Exp. 2). The total and progressive motility and velocity were evaluated by light microscope every hour (Exp. 1) or after 3 h (Exp. 2).

### Motility

The post-thaw motility was evaluated with a light microscope for the percentage of progressively motile spermatozoa, total motility percentage and a velocity score (from 1 to 3). Motility parameters were measured with an automatic sperm analyzer (Hamilton Thorn Motility Analyzer, HTM-S, version 7.2, Hamilton Thorne Research, Beverly, MA, USA) using video taping [20]. A 7-μL semen sample was placed into a Makler chamber at a temperature of 37.1°C; 2 chambers were prepared from the same sample. The chamber was placed on the thermostatically controlled stage of the motility analyzer and video recordings made as described by *Varner et al*. [20]. When the videotapes were analyzed the analyzer settings were: frames at frame rate 20 – 25/sec, minimum contrast 8, minimum size 6, low/high size gates 0.6 – 1.5, low/high intensity gates 0.6 – 1.5, motile head size 16, non- motile intensity 371, medium VAP (average path velocity) value 30, low VAP value 10, slow cells not motile, and threshold straightness 60. The videotapes were analyzed for the level of total (TMOT) and progressive motility (PROG), VAP and percentage of rapid sperm (RAP).

### Plasma membrane integrity

Plasma membrane integrity was evaluated after thawing, using 3 methods in Exp. 1 and 5 methods in Exp. 2: 1) carboxyfluorescein diacetate/propidium iodide (CFDA/PI) staining and counting of cells with a fluorescence microscope, 2) PI staining and measurement with a fluorometer (Fluoroscan Ascent, Thermo Electron Inc., Milford, MA, USA), 3) resazurin reduction test with a fluorometer, 4) HOST and counting cells with a microscope (only in Exp. 2) and 5) HOST using a fluorometer (only in Exp. 2). In Exp. 1, the tests were performed once immediately after thawing, while in Exp. 2 they were repeated after a 3-h incubation.

For evaluation of plasma membrane integrity with CFDA/PI staining, the semen was extended with a skim milk extender [19] to a concentration of 50 × 10^6 ^spermatozoa/mL. Aliquots of 20 μL of CFDA stock solution consisting of 0.46 mg CFDA in 1 mL of DMSO (dimethylsulpfoxide) and 10 μl of PI stock solution (0.5 mg PI in 1 mL of 0.9% NaCl solution) were taken, mixed with 950 μl of semen, and incubated for 8 min at 30°C [21]. A 5-μL drop was placed on a slide and overlaid with a cover slip. The proportion of fluorescent cells was counted from 200 cells in a fluorescence microscope (Olympus BH2 with epifluorescence optics, Olympus Optical Co., Tokyo, Japan) using oil immersion and a fluorescein filter set.

The second plasma membrane viability test was performed using an automatic fluorometer (Fluoroscan Ascent, Thermo Electron Inc., Milford, MA, USA), which reads a 96-well microtitration tray and has an incubation compartment. The interference filter at the excitation path and that of the emission filter showed maximum transmission at 544 nm and 590 nm, respectively. For the fluorometric assay, 20 mg of PI was dissolved in 1 L of Beltsville Thawing Solution (BTS) (USDA, Beltsville, MD, USA) and dispensed in 3-mL aliquots. Equal aliquots (50 μL) of BTS diluted semen sample (80 × 10^6 ^spermatozoa/mL) and PI solution were dispensed into a well and shaken gently for 2 min. Spermatozoa from the same samples were killed by unprotected rapid freezing and slow thawing to obtain internal control samples consisting of only non- viable cells (100% fluorescence). The control sample was immersed in liquid nitrogen for 1 min and thereafter allowed to stand at room temperature for 30 sec and then 3 min in a water bath (37°C). Blanks containing 50 μl of diluted extender and 50 μl of PI were analyzed separately for every experiment in 4 replicates; the incubation time was 8 min. The percentage of fluorescence was calculated from the ratio of fluorescence intensities of the rapidly frozen control sample and the sample to be analyzed, after comparing with the blank values [22].

### Resazurin reduction test

For the resazurin reduction test, 400 mg of resazurin was dissolved in 1 L of distilled water. One part of this solution and 9 parts of 0.9% NaCl were mixed [23]. An equal volume of this mixture and diluted sperm were combined and shaken for 2 min, then incubated for 30 min at 34°C and measured with the fluorometer, using the same fluorometer settings as in the plasma membrane viability test.

### HOST

For the HOST, semen was extended to 4 × 10^6 ^spermatozoa/mL. The hypo-osmotic solution was prepared by dissolving 1.352 g fructose and 0.735 g Na-citrate to distilled water (150 mOsm, pH 7.2). An aliquot of 0.125 mL of sperm was added to 0.5 mL of solution and the mixture was incubated for 30–45 min at 37°C. A 5-μL drop was placed on a slide and overlaid with a cover slip. A total of 200 spermatozoa per sample were evaluated for the presence of bent tails in light microscopy [24] and also analyzed with an automatic fluorometer. For fluorometric determination of the HOST, 0.5 mL of the same hypo-osmotic solution (100 mOsmol/kg) were mixed with 0.125 mL of skim milk-extended semen (concentration 100 × 10^6 ^spermatozoa/mL). The fluorometric method was the same as for PI-stained semen. The mixture was incubated at 37°C and analyzed again after 3 h.

### Morphology and bacteriology

The frozen-thawed semen smears were air-dried and stained with Giemsa according to *Watson *[25]. A total of 100 spermatozoa were evaluated with light microscopy, magnification × 1250, for major abnormalities (underdevelopment, acrosomal granules, other major acrosomal defects, diadem effects, tails bent under the head, dag effects, mid piece defects, and proximal droplets) and minor abnormalities (bent tail, twisted tail, loose normal heads, large heads, loose acrosomes, and mild acrosomal abnormalities) according to *Blom *[26].

The bacterial culture was performed by spreading a drop of each sample onto half a blood agar plate, using a 10-μL sterile loop. After incubation for 24 and 48 h at 37°C, colony forming units (CFUs) were counted and bacterial species recognized. If more than 100 CFUs were detected per sample, the number was not calculated further.

### Statistical methods

Pearson and Spearman correlation coefficients were used to study the association between the parameters. The results were accounted for, if both correlation coefficients were congruent. P-values < 0.05 were considered significant. The results were expressed as mean ± the standard error of the mean (s.e.m.). The stallions were divided into 2 groups: foaling rate of mares < 60% or > 60%. The independent sample t-test was used to test differences in the laboratory test parameters between the 2 groups of stallions. Statistical analysis was also performed separately from the material restricted to those stallions having > 20 mares (19 stallions in Exp. 1 and 16 in Exp. 2).

## Results

### Experiment 1

The percentage of normal spermatozoa varied from 51% to 89%. Major abnormalities accounted for 9.5%, including head abnormalities in 4.1% (1–12%), tail bent under the head 2.5% (0–6%), and mid piece defects 2.4% (0–7%); minor abnormalities comprised 10.7% (3–31%) including mainly bent tails 6.9% (1–29%), normal loose heads 1.4% (0–7%), and loose acrosomes 2% (0–5%). There was no association between morphological findings and foaling rate.

A total of 52% of the samples showed no microbial growth, in 41% < 100 CFUs per plate were detected, and in 7% > 100 CFUs per plate. The microbes were mainly coagulase-negative staphylococci or belonged to the families *Enterococcus*, *Enterobacteriaceae*, or *Corynebacteriaceae*. There was no association between bacteriological findings and foaling rate.

**Figure 1:**
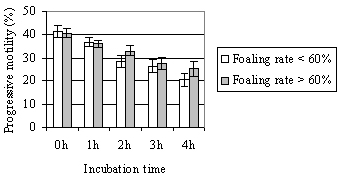
(Exp. 1) Mean (± s.e.m.) progressive motility in light microscopy during 4-h incubation in stallion groups with foaling rates of < 60% or > 60%. Number of mares per stallion was > 7.

**Figure 2:**
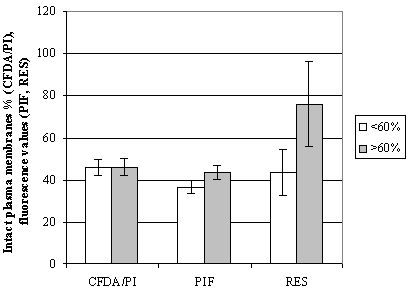
(Exp. 1) Mean (± s.e.m.) plasma membrane integrity parameters in stallion groups with foaling rates of < 60% or > 60%. Number of mares per stallion was > 7. CFDA/PI = plasma membrane integrity using light microscopy; PIF = plasma membrane integrity with PI staining using a fluorometer; RES = plasma membrane integrity with resazurin reduction test using a fluorometer.

Average CASA motility and s.e.m. were as follows: TMOT 37.0 ± 3.3, PROG 27.6 ± 2.7, and VAP 58.7 ± 2.0; these values did not correlate with fertility. Average progressive motility evaluated in light microscopy showed following changes during incubation: 0 h 40.2 ± 1.7 (min 10, max 60), 1 h 35.0 ± 1.4 (5, 50), 2 h 29.0 ± 1.6 (10, 40), 3 h 24.9 ± 1.7 (10–40), and 4 h 21.0 ± 1.7 (5–40). Progressive motility correlated significantly with foaling rate after 2–4 h of incubation (correlation coefficients 0.39 – 0.51; p < 0.05). Stallions (> 7 mares) with foaling rates of > 60% appeared to retain sperm motility slightly better than stallions with foaling rates of < 60%, although the difference was not statistically significant (Fig. 1). Similarly, semen resulting in foaling rates of > 60% showed higher plasma membrane integrity percentages measured with fluorometer than semen resulting in foaling rates of < 60%, but the differences were not statistically significant (Fig. 2).

**Figure 3:**
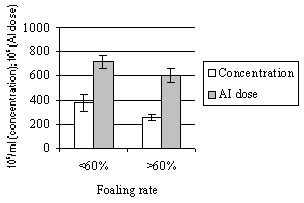
(Exp. 1) Mean (± s.e.m.) sperm concentration and total number of sperm in an AI dose in stallion groups with foaling rates of < 60% or > 60%. Number of mares per stallion was > 7.

Sperm concentration and the total number of sperm in an AI dose showed huge variation: the average concentration ± s.e.m. 383.2 ± 48.6, min 45, max 1593, and the average number of sperm/AI dose 713.2 ± 47.2, min 302, max 1777. When stallions having > 20 mares were analyzed, the total number of sperm in an AI dose showed a significant negative correlation of 0.58 with foaling rate (p < 0.05). The total number of sperm/AI dose and sperm concentration for stallion groups having foaling rates < 60% or > 60% are shown in Fig. 3, the difference being significant (p < 0.05). For all other parameters correlation coefficients with fertility were low and non-significant.

When the various parameters were compared with each other, all motility parameters correlated significantly with each other (correlation coefficients varied from 0.44 to 0.81), similarly the plasma membrane integrity tests showed significant correlations between each other (0.37 – 0.83). CFDA/PI staining with light microscopy and with a fluorometer correlated significantly also with progressive motility before incubation. The total number of sperm/AI dose showed a significant negative correlation with the other parameters, except with progressive motility during incubation (3–4 h) and CFDA/PI with light microscopy.

### Experiment 2

**Figure 4:**
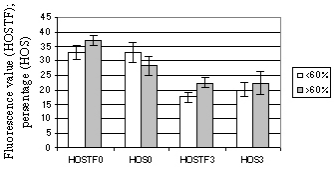
(Exp. 2) Mean (± s.e.m.) percentage of sperm positive for hypo-osmotic swelling test (HOST) in stallion groups with foaling rates of < 60% or > 60%. Number of mares per stallion was > 20. HOSTF0 = HOST with fluorometer after 0-h incubation; HOSTF3 = HOST with fluorometer after 3-h incubation; HOS0 = HOST with light microscopy after 0-h incubation; HOS3 = HOST with light microscopy after 3-h incubation.

**Figure 5:**
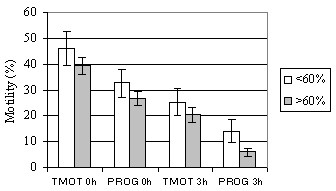
(Exp. 2) Mean (± s.e.m.) total (TMOT) and progressive motility (PROG) immediately after thawing and after 3-h incubation in stallion groups with foaling rates of < 60% or > 60%. Number of mares per stallion was > 7.

The average HOST-values were 30.1 ± 1.6 (18–49) before incubation and 21.7 ± 1.6 (9–46) after 3 h of incubation. A significant correlation coefficient of -0.50 with foaling rate (p < 0.05) was demonstrated before incubation. The average CFDA-values obtained in microscopy were 42.9 ± 2.4 (14–66) before incubation and 33.0 ± 1.8 (12–48) after the 3-h incubation. When stallions having > 20 mares were analyzed, CFDA/PI staining with light microscopy at 0-h incubation and HOST with fluorometer after a 3-h incubation showed correlation coefficients of 0.5 with foaling rate (p > 0.05). The HOST results in 2 stallion groups divided by their foaling rates are shown in Fig. 4. For other tests, correlation coefficients with foaling rate were low and non-significant. The TMOT and PROG values for stallions with foaling rates < 60% and > 60% are shown in Fig. 5.

When the various parameters were compared, TMOT, PROG, VAP, and RAP correlated after the 0-h and 3-h incubations, correlation coefficients ranging from 0.5 to 0.8. CFDA, HOST and resazurin both by microscopy and fluorometer correlated after the 0-h and 3-h incubations with coefficients of 0.4 – 0.8, but no correlation was demonstrated between these parameters and parameters depicting motility. Before incubation, the concentration showed a significant negative correlation with CFDA/PI staining, using both light microscopy and the fluorometer, but this correlation disappeared after the 3-h incubation.

## Discussion

No single test was found to be a consistently reliable method for predicting the fertilizing capacity of semen. The results of Exp. 1 could not be repeated, although the stallions and methods used were partly similar. Differences in the outcome of these experiments could be explained also by different ejaculate batches.

### Plasma membrane integrity tests

Some tendency for the plasma membrane integrity tests to be indicators of higher foaling rates was noted in our study. Fluorometers have not been much used in the examination of stallion semen. Although no strong correlation with foaling rate was found, the various fluorometric measurements did correlate with plasma membrane integrity in light microscopy and with each other. The use of fluorometers has some advantages in comparison to the use of light microscopy: it requires less time, and more cells in the sample are assessed [14]. In boars [27] and bulls [28], fluorometric measurements significantly correlated with fertility parameters.

*Neild et al*. [29] found no significant connection between the HOST and fertility but a tendency for the HOST to correlate with the number or services per pregnancy. We detected both negative and positive correlations, suggesting that this test is not suitable for evaluation of frozen-thawed stallion semen.*Nie et al*. [30] reported lower pregnancy rates for HOS+ group. *Blach et al*. [12] found indirect evidence that many immotile spermatozoa possessed an intact plasma membrane, which would indicate that these two parameters do not correlate with each other. In our first experiment, plasma membrane integrity with light microscopy correlated with many other parameters, including motility. This is in disagreement with the study of *Samper *[13] who noted membrane integrity to show extremely poor correlation with motility, particularly in preserved semen. Likewise, in our second experiment correlation between motility and plasma membrane integrity was not observed.

### Motility

Motility correlated with fertility in our first experiment but not in the second. One explanation can be pre-selection of semen. Most laboratories use 30% as a cut-off value when accepting semen for sale. The most important task of semen tests is to exclude semen of inferior quality, and this had already been done in the laboratories which had exported the semen. Another explanation is the use of different batches from several ejaculates from each stallion, but still the question on the accuracy of motility evaluation as a means of predicting fertility is raised. Semen from a stallion with the highest progressive motility (60%) did not produce any pregnancies in 10 inseminated mares. Motility has been claimed by other investigators to be a poor predictor of pregnancy rates [6,31]. However, *Voss *and his coworkers [6] suggested that although the relationship between motility and fertility is poor in the stallion, spermatozoal motility and the quality of motility are still the most reliable estimates of fertility in practice. On the other hand, *Jasko et al*. [32] reported significant correlations between motility parameters and fertility.*Newcombe *[33] reported that pregnancies per insemination decreased when semen with low motility was used. VAP is not reliable or repeatable according to *Kirk et al*. [34]. The motility results immediately after thawing do not necessarily predict the results after incubation, as was seen with progressive motility in our first experiment. Longevity tests may therefore be useful in assessing viability of semen, although *Voss et al*. [6] suggested that longevity may be of limited value in predicting potential fertility. Despite these inconsistencies between studies, motility continues to have value as an easy and economical way for estimating relative cell health.

### Sperm numbers and concentration

The total number of sperm/AI dose and concentration correlated negatively with many parameters. This can be explained by the tendency to increase AI dose and thus the concentration – the volume of one straw is limited – when low post-thaw motility is detected to increase the number of progressively motile spermatozoa and consequently the possibility of pregnancy [35]. This practice is only effective to a certain point; *Amann *[11] presented a dose-response curve showing that fertility ceases to improve as the critical number of spermatozoa needed for maximum fertility of a given male has been reached. High doses and concentration may even decrease fertility. High sperm numbers and concentration provoked more intense inflammatory responses in the uterus [36,37]. Spermatozoa at higher AI doses arrived in the oviducts later than spermatozoa contained in smaller doses [37]. Fertility dropped when the AI dose contained > 900 × 10^6 ^frozen sperm [35].

### Morphology

In our study, the percentage of morphologically normal semen and foaling rates did not correlate, perhaps because semen with a high percentage of morphological abnormalities is not frozen. *Jasko et al*. [38,32] showed the percentage of normal sperm to have a positive correlation with fertility. However, *Voss et al*. (1981) noted considerable variation in the morphologic characteristics among stallions and among ejaculates within stallions. They suggested that spermatozoal morphology may not be as valuable in evaluation of potential fertility in the stallion as it is in other large domestic animals.

### Problems of fertility studies in horses

Problems confronted in previous fertility studies are also obvious here. The number of mares inseminated per stallion was small, the straws were not from the same batch, foaling rates were collected during several years – fertility of stallions can vary between years and decrease with age. Insemination conditions, veterinary skills, management of stud farms, criteria for mare selection, etc. vary considerably. Many other factors in addition to semen characteristics influence fertility. Although obtaining pregnancy rates is difficult, they are more accurate and reliable than foaling rates. However, this does not eliminate other factors affecting fertility, such as management and reproductive performance of mares, their previous reproductive status, and possible genetic factors [32]. A multi-center study by *Samper et al*. [35] summarized the major factors affecting pregnancy rates of mares bred with frozen semen: the technician, mare age and status, insemination volume, timing of insemination, and number of sperm per dose. Standardizing all these variables is clearly not possible.

Development of freezing methods for stallion semen is dependant on finding dependable correlations between laboratory tests and fertility, which appears very difficult to achieve since the results of different studies in this field tend to be contradictive. The present study was unable to address the question of which laboratory tests would accurately predict fertility of commercially produced stallion semen. Objectivity, repeatability, and accuracy are basic requirements for laboratory assays, but many semen analysis tests do not meet these requirements [7]. Quality control of cryopreserved stallion semen remains to be a problem in practice where e.g. flow cytometry is not available. For practical purposes, it would be most important to identify semen samples that are likely to have poor fertilizing potential [4]. *Nie et al*. [30] concluded that evaluating fresh spermatozoa offered no advantage for pregnancy over simply inseminating with spermatozoa not selected for any particular characteristics.

The constraints in horse breeding – small numbers of mares per ejaculate and per stallion and the tremendous variations in mare management and insemination – never allow us to carry out trials similar to what the cattle industry has done in developing freezing methods and AI techniques. Fertility trials of horses are bound to be of little value because of these reasons [16].
